# The Antibacterial Efficacy of Far-UVC Light: A Combined-Method Study Exploring the Effects of Experimental and Bacterial Variables on Dose–Response

**DOI:** 10.3390/pathogens13080698

**Published:** 2024-08-19

**Authors:** David T. Griffin, Terence Gourlay, Michelle Maclean

**Affiliations:** 1 Department of Biomedical Engineering, University of Strathclyde, Wolfson Building, 106 Rottenrow, Glasgow G4 0NW, UK; david.t.griffin@strath.ac.uk (D.T.G.); terence.gourlay@strath.ac.uk (T.G.); 2 The Robertson Trust Laboratory for Electronic Sterilisation Technologies (ROLEST), Department of Electronic & Electrical Engineering, University of Strathclyde, Royal College Building, 204 George St, Glasgow G1 1XW, UK

**Keywords:** Far-UVC light, 222 nm, ultraviolet radiation, bacterial inactivation, transparent liquid suspension, ESKAPE pathogens, environmental decontamination

## Abstract

Far-ultraviolet C light, with a wavelength of 200–230 nm, has demonstrated broad-spectrum germicidal efficacy. However, due to increased interest in its use as an alternative antimicrobial, further knowledge about its fundamental bactericidal efficacy is required. This study had two objectives. Firstly, it investigated experimentally the Far-UVC dose–response of common bacteria suspended at various cell densities in transparent buffer, ensuring no influence from photosensitive suspending media. Increasing doses of Far-UVC were delivered to *Enterococcus faecium*, *Escherichia coli*, *Pseudomonas aeruginosa* and *Staphylococcus aureus* in PBS at 10^1^, 10^2^, 10^3^, 10^5^ and 10^7^ CFU·mL^−1^, with surviving colony-forming units enumerated (*n* ≥ 3). Secondly, through a systematised literature review, this work sought to explore the impact of genus/species, Gram type, cell form, cell density and irradiance on dose–response. The screening of 483 publications was performed with 25 included in the study. Data for 30 species were collated, analysed and compared with the experimental results. Overall, Gram-positive species showed greater resilience to Far-UVC than Gram-negative; some inter-species and inter-genera differences in resilience were identified; endospores were more resilient than vegetative cells; the results suggested that inactivation efficiency may decrease as cell density increases; and no significant correlation was identified between irradiance and bactericidal dose effect. In conclusion, this study has shown Far-UVC light to be an effective decontamination tool against a vast range of bacterial vegetative cells and endospores.

## 1. Introduction

Far ultraviolet C (Far-UVC) describes wavelengths between 200 and 230 nm [1,2], a range with proven germicidal efficacy against a wide range of pathogens including bacteria [3,4], viruses [5,6] and fungi [4,7]. In recent years, interest in the use of antimicrobial Far-UVC for a diverse range of applications has grown, including the decontamination of healthcare facilities and equipment [8,9], drinking water [10,11,12] and indoor air [1,13,14]. This growth has coincided with increased evidence indicating its superior safety for mammalian cell exposure over alternative UVC wavelengths [15,16,17,18,19], a result of its attenuation before reaching the cell nucleus [20]. It has also coincided with the ongoing severe acute coronavirus (SARS-CoV-2) pandemic [21], which has renewed focus on the need for effective environmental decontamination technologies [1,22,23,24].

While the concerns presented by the SARS-CoV-2 pandemic continue, so too does the burden presented by bacterial pathogens. As the pathogen form responsible for most hospital-associated infections [25] and between approximately 60 and 90% of sepsis infections [26,27,28,29], bacteria present a considerable drain on global healthcare resources. Furthermore, the growing concern of antimicrobial resistance (AMR) amplifies the existing threat from bacteria. For example, through a systematic review, Murray et al. [30] estimated that 1.27 million deaths globally could be attributed to bacterial AMR in 2019 alone. Given the threat to life, as well as the limiting effect AMR could have on the development of new therapeutic and diagnostic practises due to fears of infectious complications [31], novel bactericidal technologies as alternatives to antibiotics are paramount to continued medical progress. Consequently, it is likely that Far-UVC use in the fields of environmental and biomedical decontamination will continue to grow.

Practical decontamination applications involve the inactivation of pathogens in a variety of situations. Suspending media can range from transparent or semi-transparent liquids to more complex biological or organic matrices. These include environmental treatment applications, such as drinking and wastewater decontamination [10,12,32] as well as biomedical applications requiring the decontamination of bodily fluid, e.g., blood plasma in transfusion medicine [33]; saliva in endotracheal intubation [34]; and urine in urethral catheters [35]. However, since Far-UVC light technologies are in their relative infancy, few comparative data exist on bacterial inactivation using these wavelengths.

In response to this gap in knowledge, this study had two overarching objectives. Firstly, it investigated experimentally the Far-UVC dose–response of some common pathogenic bacterial species, suspended at a range of densities, in phosphate-buffered saline (PBS), a common transparent and minimal medium. The use of this minimal medium enabled the fundamental antibacterial efficacy of Far-UVC light without influence from photosensitive components in the suspending medium to be determined. The second objective was to perform a thorough comparison of the experimental findings with all relevant published data on the topic. To do this, a systematised review of the existing literature was conducted, allowing the effect on dose–response of bacterial variables (*genus*/*species*, *Gram type* and *cell form*) and experimental variables (*cell density* and *irradiance*) to be determined. Furthermore, this study is intended to aid other researchers by providing a basic hierarchical reference index of fundamental bacterial inactivation by species using Far-UVC light.

## 2. Materials and Methods

### 2.1. Experimental Methods

#### 2.1.1. Far-UVC Light Source

**Figure 1:**
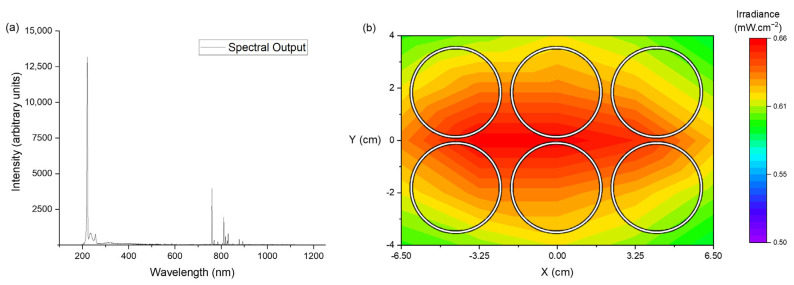
Optical profile of the Unilam Kr-Cl excimer lamp. (**a**) Emission spectrum from the lamp with the power supply set to 180 V, captured using HR4000 spectrometer. Peak emission at 220.97 nm. (**b**) Irradiance map at 35 cm from Kr-Cl surface over the surface area of a 6-well plate (with the plate outline superimposed on the irradiance map). Irradiance values account for a reduction in transmission caused by a quartz window which covered sample plates during irradiation.

A single krypton-chloride (Kr-Cl) excimer lamp and power supply, with an input voltage of 180 V, was used (EX 240S10-222 and PS-EX0K35/EAI/P/DI-F, respectively; Unilam Co. Ltd., Ulsan, Republic of Korea). The peak emission wavelength was 220.97 nm (Figure 1a) with a bandwidth of 3.66 nm at full width half maximum, recorded using an HR4000 spectrometer (Ocean Optics, Ostfildern, Germany) and Spectra Suite (v. 2.0.151) software. The experimental setup was housed in a fume cupboard due to the potential for ozone generation [36]. The lamp was clamped in position 35 cm above the irradiated sample surface, and the optical irradiance profile was mapped over a surface area of 13 × 8 cm, using a Nova power meter (Ophir Spiricon Europe GmbH, Darmstadt, Germany). The mean irradiance over the irradiated area (Figure 1b) was 0.62 ± 0.04 mW·cm^−2^, which includes an 8.6% loss in irradiance due to a quartz window used to cover the sample plates during irradiation. The applied dose was then calculated according to Equation (1):

(1) $${Dose\;(mJ\cdot cm}^{-2})=Time\left(s\right)\times Irradiance\;{(mW\cdot cm}^{-2})$$

#### 2.1.2. Preparation of Bacterial Suspensions

Four bacterial strains were used experimentally in this study: *Enterococcus faecium* LMG 11423, *Escherichia coli* NCTC 9001, *Pseudomonas aeruginosa* NCTC 9009 and *Staphylococcus aureus* NCTC 4135. Bacteria were cultured under rotary conditions (37 °C; 18–24 h; 120 rpm) in 100 mL nutrient broth (Oxoid Ltd., Basingstoke, UK), with the exception of *E. faecium*, which was cultured in tryptone soya broth (Oxoid Ltd., Basingstoke, UK). Broths were centrifuged (10 min at 3939× *g*), with the cell pellet then resuspended in 100 mL phosphate-buffered saline (PBS), and serially diluted in PBS to the required cell densities for Far-UVC exposure.

#### 2.1.3. Far-UVC Irradiation of Bacteria in Liquid Suspension

Bacterial suspensions of 1.4 mL volume were transferred into the wells of a 6-well plate, providing a depth of ≈1.5 mm. The sample plate was covered with a quartz window and placed on an orbital shaker (100 rpm) positioned 35 cm below the Kr-Cl lamp. The orbital shaker ensured the constant mixing of the sample while also minimising the variance in irradiance across the irradiated wells. Samples were then irradiated for increasing time periods/doses (*n* = 3). A FLIR ONE Pro thermal imaging camera (Teledyne Flir, Kent, UK) was periodically used to ensure irradiated samples did not exceed room temperature during irradiation.

Following light exposure, samples (*n* ≥ 3) were plated onto nutrient agar (or tryptone soya agar for *E. faecium*; Oxoid Ltd., Basingstoke, UK), using standard microbiological plating techniques. If counts were expected to be ≥10^3^ colony forming units per millilitre (CFU·mL^−1^), samples were serially diluted and spread-plated (50 or 100 µL volumes, giving a detection limit of 20 or 10 CFU·mL^−1^, respectively); if counts were expected to be ≤10^2^ CFU·mL^−1^, samples were pour-plated (one millilitre volumes, giving a detection limit of one CFU·mL^−1^).

Sample plates were incubated for 18–24 h at 37 °C before manual counting on a colony counter (Stuart SC6 Plus Colony Counter; Cole-Palmer, Cambridgeshire, UK). Results were recorded in CFU·mL^−1^. To enable an effective comparison of Far-UVC dose–responses, the 1-Log_10_ inactivation dose (1-Log_10_ ID) (mJ·cm^−2^) was calculated for all dose–response curves using Equation (2). This was carried out following the method outlined by Tomb et al. [37] in which the highest reported dose for the greatest significant inactivation was recorded. Where there was evidence of a prominent tailing of inactivation as the dose increased, the dose and relative inactivation prior to this tailing was recorded instead.

(2) $$1-{Log}_{10}ID=\frac{applied\;dose}{mean\;{Log}_{10}\;inactivation\;at\;applied\;dose}$$

### 2.2. Literature Review

Three databases were selected for searching as part of the systematised literature review: Scopus, Pubmed (which includes Medline) and Compendex. A single research question was then composed: ‘*How has Far-UVC light been used in the inactivation of bacteria in transparent suspension?*’. The research question was divided into three primary constituent terms, *Far-UVC light*, *Inactivation* and *Bacteria*, with an extensive list of synonyms created for each term. These synonyms were then combined to create search strings for input in the selected databases using Boolean searching. All searching was performed on 23rd February 2024. All search terms and strings are outlined in Tables S1 and S2 of the Supplementary Materials.

**Figure 2:**
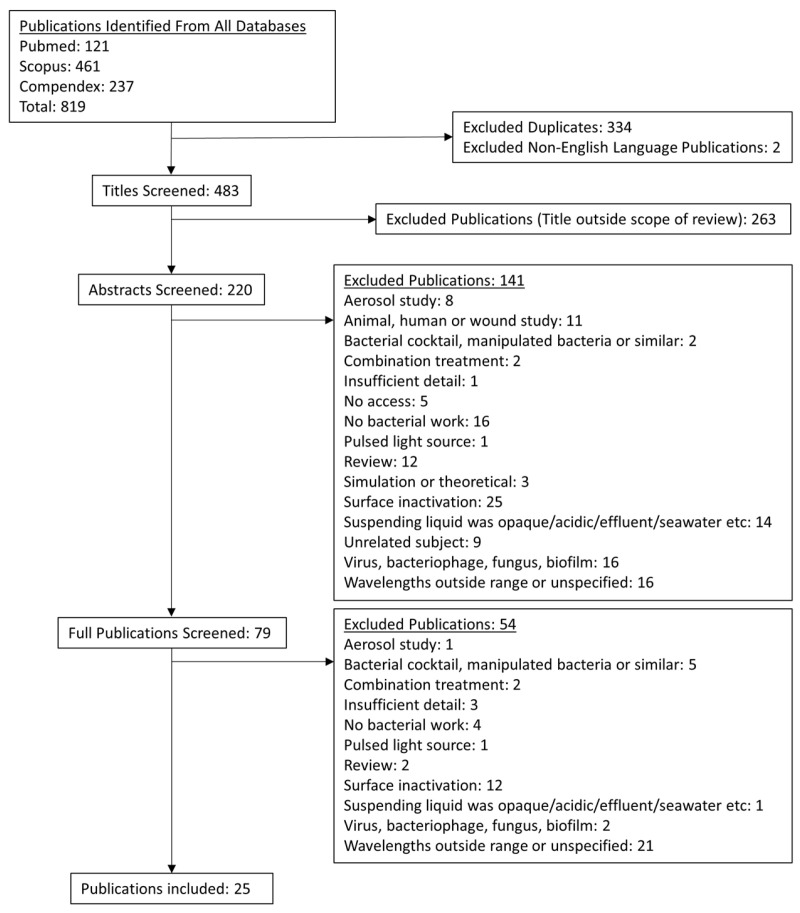
Flowchart outlining the publication selection process and the number of publications at each stage in the systematised review, based on the inclusion and exclusion criteria.

A total of 483 studies met the initial inclusion criteria across the three databases after duplicates and non-English language publications were discounted. The screening of studies through their titles, abstracts and full contents where necessary yielded 25 studies for inclusion, as outlined in the flowchart presented in Figure 2. Excluded studies included those using light sources beyond the Far-UVC range, those in which bacteria were irradiated after spreading on surfaces and those which employed nutritious or opaque suspending media. All specific inclusion and exclusion criteria are detailed in Table 1.

Data were manually collected from studies, whether documented in text or presented in tables or graphs, following the method outlined by Tomb, et al. [37]. In short, from each paper, the highest reported dose for the greatest statistically significant inactivation was recorded. In the case of data provided only graphically, pixels were digitally counted to ensure the recorded data were as accurate as possible. In the case of data with evidence of a prominent tailing of inactivation as the dose increased, the dose and relative inactivation prior to this tailing was recorded instead.

As earlier outlined, data were gathered with the goal of exploring the effects of a range of variables on Far-UVC dose–response. These variables included bacterial variables (*genus*/*species*, *Gram type* and *cell form*) and experimental variables (*cell density* and *irradiance*). Values for the experimental variables were not present in all publications intended for inclusion. Consequently, studies were included only in the categories for which all data were specified. Furthermore, to enable an effective comparison of dose–responses, the 1-Log_10_ ID value, where not specified in a publication, was calculated as outlined in the previous section, using Equation (2). For both the variables of *cell density* and *irradiance*, only species for which experimental data relating to three or more unique cell densities/irradiances were available were included. All calculations were rounded to two significant figures.

In total, data relating to the inactivation of 30 different bacterial species was extracted from 25 studies, with 322 unique data values. These data related to 10 Gram-negative and 20 Gram-positive species, three species with data for both their endospore and vegetative cell forms, seven species with data for their endospore form alone and 20 species with data for vegetative cells alone.

Data were gathered according to bacterial species; one species category could include multiple individual strains. In the case of species for which there were multiple data points retrieved for a single variable (e.g., *cell density*), the median value was calculated and used to limit the effect of potential outliers, as carried out by Hessling et al. [2]. Data for uncommon or genetically modified mutant strains were excluded due to the potential for unexpected results.

### 2.3. Statistical Analysis

For all experimental work described, a paired *t*-test was used to compare surviving bacterial populations at each dose point with their respective starting cell density to determine statistical significance. Pearson correlation values were also calculated between the surviving bacterial population and applied dose. For systematised review data, Pearson correlation values were calculated between two variables where three or more unique data points were available.

All statistical calculations were performed at the 95% confidence level using Minitab (v18) software (Minitab LLC, State College, PA, USA). All graphs were created using Origin (v2019b) software (OriginLab Corp, Northampton, MA, USA). The Pearson correlation coefficient value designations, as outlined by Mukaka [38] and shown in Table 2, were used in all cases when describing the relationship between variables.

When describing some values, the term mean* has been used. This term describes the mean of a collection of median values for a specific variable for a collective group. For example, the mean* Far-UVC 1-Log_10_ ID for Gram-negative vegetative cells was calculated by determining the average of the median 1-Log_10_ ID values for the vegetative cells of all Gram-negative species for which data were available.

## 3. Results

### 3.1. Experimental Analysis of the Germicidal Efficacy of Far-UVC Light

**Figure 3:**
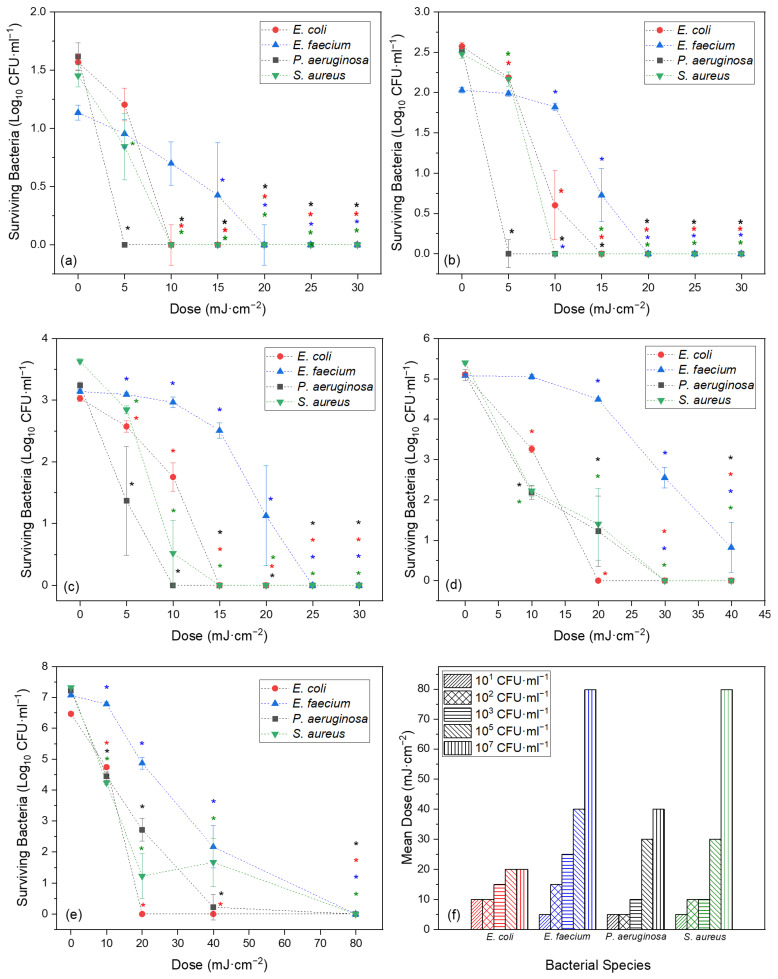
Bacterial inactivation of *E. coli*, *E. faecium*, *P. aeruginosa* and *S. aureus* suspended in PBS in cell densities of (**a**) 10^1^, (**b**) 10^2^, (**c**) 10^3^, (**d**) 10^5^ and (**e**) 10^7^ CFU·mL^−1^, using increasing doses of Far-UVC light and an irradiance of 0.62 mW·cm^−2^ (*n* ≥ 3 ± SD; detection limit ≤20 CFU·mL^−1^); * denotes significant inactivation compared with its respective starting population (paired *t*-test, *p* < 0.05). (**f**) Comparison of the dose required to achieve near complete inactivation (<20 CFU·mL^−1^ surviving) for each bacterial species for each cell density.

The following sections detail the experimental results for the inactivation of *E. coli*, *E. faecium*, *P. aeruginosa* and *S. aureus* while suspended in PBS at a range of cell densities (10^1^ to 10^7^ CFU·mL^−1^), using Far-UVC light at a single irradiance of 0.62 mW·cm^−2^ (Figure 3).

#### 3.1.1. Effect of Cell Density on Far-UVC Bactericidal Efficacy

In cell densities of 10^1^, 10^2^ and 10^3^ CFU·mL^−1^, there was a notable reduction in the number of viable bacteria for all four species with exposure to the minimum dose of 4.99 mJ·cm^−2^ (Figure 3a–c). This reduction was most pronounced in *P. aeruginosa*, with this minimum dose achieving ≥98.65% inactivation from all three cell densities (*p* ≤ 0.021). This reduction reached statistical significance by a dose of 9.99 mJ·cm^−2^ for *E. coli*, *P. aeruginosa* and *S. aureus* (*p* ≤ 0.021), with ≥94.70% inactivation achieved in all three species. However, for a statistically significant reduction to be achieved in *E. faecium* across all three cell densities, a dose of 14.98 mJ·cm^−2^ was required, achieving ≥76.68% inactivation (*p* ≤ 0.003).

*Enterococcus faecium* was consistently the most challenging species to inactivate across all three cell densities. For example, in a cell density of 10^1^ CFU·mL^−1^, complete measurable inactivation was achieved at or below 14.98 mJ·cm^−2^ in all other species. However, in *E. faecium*, this dose only achieved 80.49% (*p* = 0.003) inactivation, with a dose of 29.96 mJ·cm^−2^ needed to achieve complete measurable inactivation (*p* = 0.008).

Generally, as the cell density increased, so too did the dose required to achieve complete or near complete measurable inactivation (Figure 3f). For example, in a cell density of 10^1^ CFU·mL^−1^, *P. aeruginosa* had reached complete measurable inactivation by the minimum dose of 4.99 mJ·cm^−2^. In a cell density of 10^3^ CFU·mL^−1^, however, this was not achieved until 9.99 mJ·cm^−2^. Similarly for *S. aureus*, in a cell density of 10^1^ CFU·mL^−1^, complete measurable inactivation was achieved at a dose of 9.99 mJ·cm^−2^ (*p* = 0.015), but 14.98 mJ·cm^−2^ was required to achieve this in a cell density of 10^3^ CFU·mL^−1^ (*p* < 0.001).

In the higher cell densities of both 10^5^ and 10^7^ CFU·mL^−1^ (Figure 3d,e), reductions in viable bacteria were achieved at the minimum dose of 9.99 mJ·cm^−2^ for all four species. This was statistically significant (*p* < 0.001) for all species, except *E. faecium*, which required a dose of 19.97 mJ·cm^−2^ in a cell density of 10^5^ CFU·mL^−1^ to reach significant inactivation (74.11% inactivation; *p* < 0.001).

While relatively similar dose–responses were recorded for *E. coli*, *P. aeruginosa* and *S. aureus*, *E. faecium* was again the most resilient species to inactivate overall. At the minimum dose of 9.99 mJ·cm^−2^, ≥98.58% inactivation was achieved in *E. coli*, *P. aeruginosa* and *S. aureus* (*p* ≤ 0.001) in cell densities of 10^5^ and 10^7^ CFU·mL^−1^, yet only 48.52% inactivation was achieved in *E. faecium* in 10^7^ CFU·mL^−1^ (*p* = 0.001) and an insignificant 5.81% inactivation in 10^5^ CFU·mL^−1^ (*p* = 0.505).

Similarly, a dose of 29.96 mJ·cm^−2^ achieved complete measurable inactivation in all species in a cell density of 10^5^ CFU·mL^−1^, except *E. faecium,* which failed to achieve this even at the maximum dose of 39.94 mJ·cm^−2^. At the highest cell density of 10^7^ CFU·mL^−1^, complete measurable inactivation was achieved in all species by the 79.88 mJ·cm^−2^ dose point (*p* < 0.001).

#### 3.1.2. Far-UVC 1-Log_10_ Inactivation Dose (ID) for Differing Cell Densities

The calculated Far-UVC 1-Log_10_ ID for each species and cell density are presented in Table 3. Across all five cell densities of 10^1^ to 10^7^ CFU·mL^−1^, the mean 1-Log_10_ ID calculated for *E. coli*. *E. faecium*, *P. aeruginosa* and *S. aureus* were 4.63, 11.03, 3.96 and 6.30 mJ·cm^−2^, respectively. When grouped according to Gram type, the Gram-positive bacteria had a mean 1-Log_10_ ID of 8.67 mJ·cm^−2^ and the Gram-negative species had a mean value of 4.29 mJ·cm^−2^.

**Figure 4:**
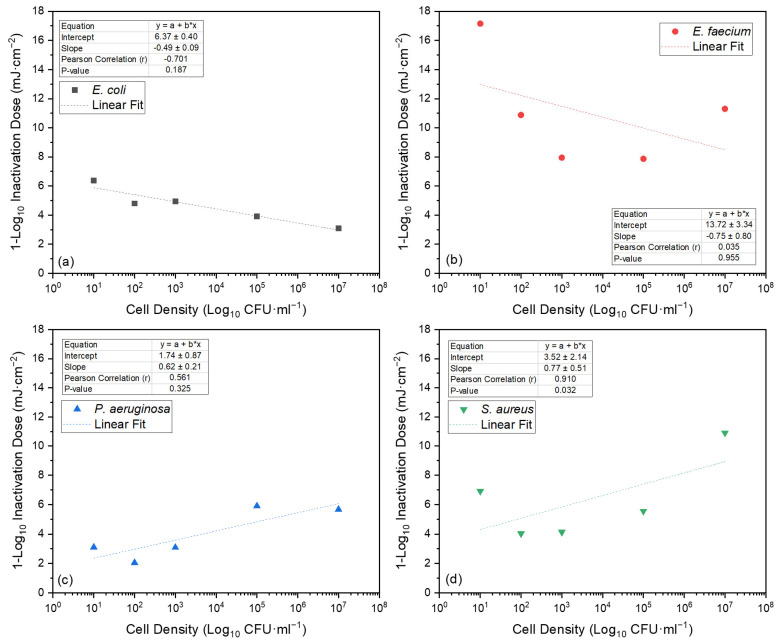
Bacterial cell density (Log_10_ CFU·mL^−1^) versus measured Far-UVC 1-Log_10_ ID for (**a**) *E. coli*, (**b**) *E. faecium*, (**c**) *P. aeruginosa* and (**d**) *S. aureus*. 1-Log_10_ ID calculated by dividing the dose (mJ·cm^−2^) at which the greatest statistically significant inactivation was achieved prior to tailing by the corresponding mean inactivation (Log_10_ CFU·mL^−1^) (based on the inactivation kinetics presented in Figure 3). Pearson correlation and *p*-values calculated using Minitab (v18); linear fit performed using Origin (v2019b).

For all four species, the identified relationships between cell density and Far-UVC 1-Log_10_ ID using the Pearson correlation are presented in Figure 4 and varied from strong negative correlation for *E. coli* to a very strong positive correlation for *S. aureus*. This relationship was statistically significant only for *S. aureus* (*p* = 0.032).

### 3.2. Literature Review

The results from analysis of the data collected through systematised literature review are detailed in the following paragraphs, according to Far-UVC *1-Log_10_ ID*, *cell density* and *irradiance*.

#### 3.2.1. Comparison of Far-UVC 1-Log_10_ Inactivation Dose

**Figure 5:**
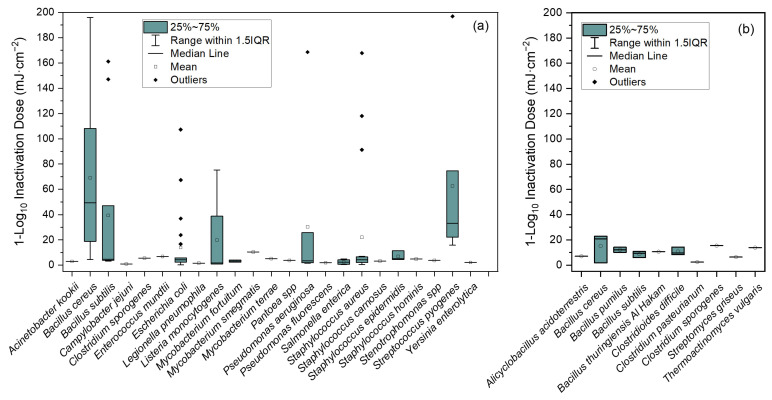
Far-UVC inactivation data collected through the systematised literature review showing 1-Log_10_ ID value (mJ·cm^−2^) for (**a**) vegetative cells and (**b**) endospores. Where 1-Log_10_ ID is needed to be calculated for an individual species, the dose (mJ·cm^−2^) at which the greatest statistically significant inactivation was achieved prior to tailing was divided by the corresponding inactivation (Log_10_ CFU·mL^−1^).

The results for the median Far-UVC 1-Log_10_ ID for all bacterial species are presented in Figure 5 and Table 4. Clear from the data presented in Figure 5 is the considerable impact made by the number of data sources available for a given species. For example, the *interquartile range* (*IQR*) and number of outliers was substantially greater for many of the species with a higher number of data points and source publications (e.g., vegetative cells of *Bacillus cereus*, *Escherichia coli* and *Staphylococcus aureus*). This is likely due to variability in study-to-study parameters, e.g., the spectral output of the light source used; the presence or absence of optical filters; differing strains of bacterial species; the specific optical properties and depth of the suspending liquid, etc.

Considering the data for vegetative cells (Figure 5a), median values ranged from a minimum 1.02 mJ·cm^−2^ for *Campylobacter jejuni* to a maximum 49.31 mJ·cm^−2^ for *Bacillus cereus*. Vegetative cells of Gram-positive species were more challenging to inactivate than Gram-negative, with a mean* 1-Log_10_ ID of 10.60 mJ·cm^−2^ for all Gram-positive species (59 data points). By comparison, for Gram-negative species, this mean* value was 2.73 mJ·cm^−2^ (40 data points). Therefore, based on the data collected, Gram-positive bacteria required 388.28% of the dose required to achieve the same level of inactivation as Gram-negative species. Within this dataset of vegetative cells alone, of the seven bacterial species with the lowest median 1-Log_10_ ID, six were Gram-negative. By contrast, the nine bacterial species with the highest median 1-Log_10_ ID were Gram-positive.

Considering the data for endospores (Figure 5b), *B. cereus* spores had the highest median 1-Log_10_ ID at 20.86 mJ·cm^−2^ (three data points). The endospores of *Clostridium pasteurianum* had the lowest median 1-Log_10_ ID at 2.40 mJ·cm^−2^ (one data point).

When the mean* 1-Log_10_ ID for all vegetative cells and all endospores was calculated, the values were found to be 7.18 mJ·cm^−2^ (99 data points) and 10.74 mJ·cm^−2^ (17 data points), respectively. Therefore, on average, endospores required 149.58% of the dose required by vegetative cells to achieve the same inactivation.

Within the review criteria, three species capable of forming endospores had data available for both their vegetative cell and endospore forms: *Bacillus cereus*, *Bacillus subtilis* and *Clostridium sporogenes*. Of these three, *B. cereus* alone was more resilient as vegetative cells than as endospores; vegetative cells of this species required 236.39% the dose required by the endospores to achieve the same 1-Log_10_ inactivation. This seeming superior Far-UVC resilience of the vegetative cell form of *B. cereus* over the endospore form is discussed at length in Section 4. For *B. subtilis* and *C. sporogenes*, by comparison, their endospore form proved more resilient to Far-UVC inactivation. Endospores of *B. subtilis* required 199.36% the dose required by their vegetative cell form to achieve the same inactivation. Endospores of *C. sporogenes* required 277.16% the dose required by their vegetative cell form to achieve the same inactivation.

#### 3.2.2. Effect of Bacterial Cell Density on Far-UVC 1-Log_10_ Inactivation Dose

**Figure 6:**
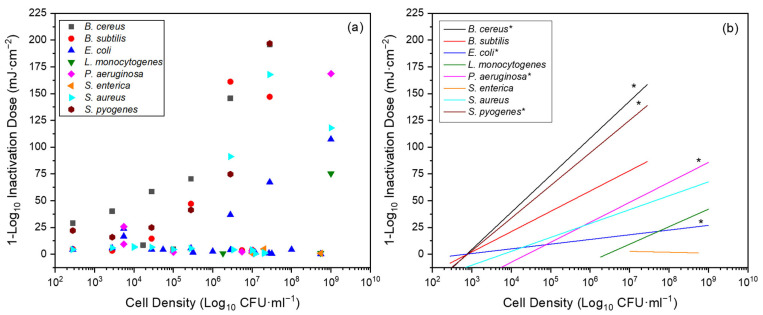
Graphs displaying data collected through systematised review for (**a**) bacterial cell density versus Far-UVC 1-Log_10_ ID for all bacterial species, with data pertaining to at least three unique cell densities; (**b**) straight lines fitted using Origin (v2019b) to demonstrate relationship between cell density and Far-UVC 1-Log_10_ ID for each individual bacterial species. * denotes significant Pearson correlation (*p* < 0.05). All bacteria are in vegetative cell form.

For the variable of *cell density*, all data are presented in Figure 6a. Specifically, the effect of this variable on the Far-UVC 1-Log_10_ ID was considered.

For vegetative cells, those species that met the criteria were the five Gram-positive *B. cereus*, *B. subtilis*, *L. monocytogenes*, *S. aureus* and *S. pyogenes* and the three Gram-negative *E. coli*, *P. aeruginosa* and *S. enterica*. There were insufficient data to explore this relationship with endospores. All correlation data are presented in Figure 6b and Table 5.

For the majority of species, there was a positive correlation between cell density and 1-Log_10_ ID. This correlation was significant in half of the cases. For four of the eight species, *B. cereus*, *L. monocytogenes*, *S. pyogenes* and *P. aeruginosa*, there was a strong or very strong positive correlation between the two variables (*p* = 0.015, 0.367, 0.001, <0.001, respectively). In all remaining cases, the correlation was also positive, except in the case of *S. enterica* (r = −0.430; *p* = 0.717). It must be noted that although the Pearson correlation was employed to analyse the relationship between the two variables, this is recommended for variables for which there are at least 30 samples; here, it has been employed for some with as few as 3 samples.

#### 3.2.3. Effect of Irradiance on Far-UVC 1-Log_10_ Inactivation Dose

**Figure 7:**
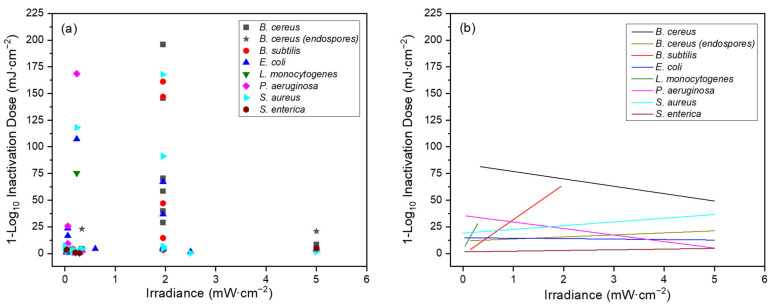
Graphs displaying data collected through systematised review for (**a**) irradiance versus Far-UVC 1-Log_10_ ID for all bacterial species with data pertaining to at least three unique irradiances; (**b**) straight lines fitted using Origin (v2019b) to demonstrate relationship between irradiance and 1-Log_10_ ID for each individual bacterial species. Significant Pearson Correlation did not result in any case (*p* < 0.05). All bacteria are in vegetative cell form unless specified otherwise.

For the variable of *irradiance*, all data are presented in Figure 7a. Specifically, the effect of this variable on the Far-UVC 1-Log_10_ ID was considered.

Across both vegetative cells and endospores, there were seven species in total, with *B. cereus* being the only species to feature in both categories. The other vegetative cells were the three Gram-positive species *B. subtilis*, *L. monocytogenes* and *S. aureus* and the three Gram-negative species *E. coli*, *P. aeruginosa* and *S. enterica*.

Data for this variable are outlined in Figure 7b and Table 6. When the correlation between 1-Log_10_ ID and the irradiance used was explored, there was no obvious relationship for most species. In seven of the eight cases, there was either a negligible or weak positive correlation between the two variables. A strong positive relationship was identified only for *S. enterica*. Significance (*p* < 0.05) was not reached in any of the eight cases.

## 4. Discussion

This study had two overarching goals. Firstly, we sought to investigate experimentally the Far-UVC dose–response of common pathogenic bacterial species from a range of cell densities suspended in a minimal medium. This would establish the fundamental efficacy of Far-UVC for bacterial inactivation. Secondly, we aimed to perform a thorough comparison of the experimental findings with all relevant published data and, in doing so, to determine how bacterial variables (genus/species, Gram type and cell form) and experimental variables (irradiance and cell density) affect that dose–response. Furthermore, this study was intended to act as a useful reference for other researchers on the topic of Far-UVC-based bacterial inactivation. The following paragraphs discuss the experimental and systematised review findings according to each of the variables involved.

### 4.1. Influence of Gram Type on Susceptibility to Far-UVC Inactivation

One common grouping method when assessing bacterial susceptibility to inactivation is Gram type. However, from an initial scoping search, the uncertainty of a Gram-type effect on Far-UVC bactericidal efficiency was evident.

In their work with foodborne pathogens, Kang et al. [43] compared the Far-UVC inactivation of two Gram-positive (*S. aureus* and *L. monocytogenes*) and two Gram-negative (*S. typhimurium* and *E. coli*) bacteria, finding the Gram-positive species to be more resistant to inactivation. Similarly, when data from Narita et al. [4] was examined, which explored the inactivation of seven Gram-positive and -negative species as vegetative cells, the two most resilient species were Gram-positive *B. cereus* and *C. sporogenes*. Yet, the second-to-least resilient species identified was the Gram-positive *S. aureus*. Furthermore, the 1-Log_10_ ID for *S. aureus* was lower than that of the Gram-negative species *S. enterica*, *E. coli* and *P. aeruginosa*. Gierke and Hessling [39] explored the use of non-pathogenic surrogates for ESKAPE pathogens with Gram-positive *E. mundtii* and *S. carnosus* and Gram-negative *A. kookii*, *P. fluorescens* and *E. coli*. They found Gram-negative *E. coli* to be the most resilient of the five species to Far-UVC inactivation, with the second and third most resilient being the Gram-positive *E. mundtii* and *S. carnosus*.

While these examples provide only a brief overview of the published data, they highlight the absence of an obvious Gram-type effect on Far-UVC inactivation. Through the combination of experimental work and a systematised review of the existing work on the topic, it was hoped that this effect could be irrefutably proved or disproved.

From the experimental work with *E. coli*, *E. faecium*, *P. aeruginosa* and *S. aureus*, there appeared to be a clear Gram-type effect. The mean 1-Log_10_ ID values for the Gram-positive and -negative species were 8.67 mJ·cm^−2^ and 4.30 mJ·cm^−2^, respectively (Table 3). From the systematised review data, for vegetative cells considered in isolation, it was found that the mean* 1-Log_10_ ID for Gram-positive species was 10.60 mJ·cm^−2^ compared with 2.73 mJ·cm^−2^ for Gram-negative species (Figure 5). As such, both result sets provide considerable evidence in favour of a Gram-type effect on Far-UVC resilience, with Gram-negative species more susceptible to inactivation.

### 4.2. Influence of Genus/Species on Susceptibility to Far-UVC Inactivation

In the experimental work, there was some variance in 1-Log_10_ ID between the four species used. While the dose–response of *E. coli*, *P. aeruginosa* and *S. aureus* was relatively similar in all experimental work, the behaviour of *E. faecium* differed considerably. Across all cell densities, *E. faecium* consistently required higher Far-UVC doses to achieve similar levels of inactivation to the other bacteria (Figure 3 and Table 3). This has also been noted in other studies where *Enterococcus* spp. have proven resilient to decontamination [61,62]. Unfortunately, the experimental work performed did not include multiple species from any single genus, excluding the possibility of any intra-genus comparison.

However, within the collected systematised review data, this was possible, and some variance in 1-Log_10_ ID was recorded (Table 4). For example, *S. epidermidis* and *S. aureus* had median 1-Log_10_ ID values of 5.14 mJ·cm^−2^ (3 data points) and 4.31 mJ·cm^−2^ (20 data points), a 16.15% reduction in the required dose to achieve the same outcome. More surprisingly, the median 1-Log_10_ ID for vegetative cells of *B. cereus* was 49.31 mJ·cm^−2^ (eight data points). However, a 90.51% reduction in dose to 4.68 mJ·cm^−2^ (10 data points) achieved the same level of inactivation in *B. subtilis*, suggesting that for some genera, a large inter-species effect may be observed.

The mean 1-Log_10_ ID values for individual species measured in the experimental work were generally comparable to the median values calculated through the systematised review (Table 3 and Table 4). For *E. coli*, the experimentally recorded mean 1-Log_10_ ID value was 4.63 mJ·cm^−2^ and the collected data median was 4.32 mJ·cm^−2^; for *P. aeruginosa,* the experimentally recorded mean value was 3.96 mJ·cm^−2^ and the collected data median was 3.43 mJ·cm^−2^; for *S. aureus,* the experimentally recorded mean value was 6.30 mJ·cm^−2^ and the collected data median was 4.31 mJ·cm^−2^. Unfortunately, *E. faecium* was not represented in the data collected within the confines of the systematised review. The closest related species with collected data was *Enterococcus mundtii*, a species from the same genus as *E. faecium*. For *E. faecium*, the experimentally recorded mean value was 11.03 mJ·cm^−2^ and the collected data median value for *E. mundtii* was 6.90 mJ·cm^−2^. In the collected data, *E. mundtii* showed the fourth highest median 1-Log_10_ ID of 23 species of vegetative cells after *Bacillus cereus*, *Streptococcus pyogenes* and *Mycobacterium smegmatis*.

These results demonstrate that some inter-genera and inter-species differences exist in bacterial response to Far-UVC irradiation. This is unsurprising, given the morphological and biochemical variation across genera and species [63]. However, they also demonstrate that the majority of species can be effectively inactivated by relatively low, common Far-UVC doses: for all vegetative cells, the mean* 1-Log ID of 7.18 mJ·cm^−2^ would achieve ≥1-Log_10_ inactivation in 87% of the species included in this review; similarly for endospores, the mean* 1-Log_10_ ID of 10.74 mJ·cm^−2^ would achieve ≥1-Log_10_ inactivation in 60% of all species included.

### 4.3. Influence of Cell Form on Susceptibility to Far-UVC Inactivation

With regard to cell form, the experimental work involved only vegetative cells and no endospores. However, both forms were included in the systematised review of literature. Rather unsurprisingly, endospores prove challenging to inactivate using Far-UVC. For example, when both vegetative cells and endospores were considered side by side by Narita et al. [4], they found *B. cereus* and *C. sporogenes* endospores to be the most resilient of 10 cell and endospore types tested. In fact, when ranked in terms of inactivation resilience, three of the top four places were occupied by endospores, with only the vegetative form of *B. cereus* displacing endospores of *C. difficile* from the third position. Similarly, when data from Taylor et al. [3] was scrutinised, of the nine cell and endospore types included, the four highest 1-Log_10_ IDs were for endospores.

The data from the systematised review broadly supported this conclusion, with the mean* 1-Log_10_ ID for endospores 149.58% that of the vegetative cells. This generally held true also for species for which the data were collected for both the endospore and vegetative cell form, with two of the three species proving more resilient as an endospore than a vegetative cell.

However, notably for *B. cereus,* the vegetative cell form was found to be substantially more resilient than its endospore form, with median 1-Log_10_ ID values of 49.31 and 20.86 mJ·cm^−2^, respectively. This was a very surprising result, given that previous research has found spores of *Bacillus* species to be between 5 and 50 times more resilient to UV radiation than their vegetative counterparts [64]. However, when the contributing papers were scrutinised further, it was discovered that one publication in particular had a substantial impact on this median value. Matafonova et al. [40], using vegetative cells of *B. cereus*, *B. subtilis*, *E. coli*, *S. aureus* and *S. pyogenes* at a range of cell densities from 10^2^ to 10^7^ CFU·mL^−1^, found that cell densities at 10^6^ and 10^7^ CFU·mL^−1^ resulted ‘in a higher tailing plateau and nonlinear survival curves’ [40] (p. 512). That is, the rate of inactivation was particularly low and, in some cases, ≥10^2^ CFU·mL^−1^ surviving bacteria remained, even at doses approximating 585 mJ·cm^−2^. Moreover, in some cases, the doses required by Matafonova et al. [40] at lower cell densities were considerably higher than those published elsewhere to achieve comparable inactivation. While the work of Matafonova et al. [40] met all inclusion criteria, it had a substantial effect on the 1-Log_10_ ID value for vegetative cells of *B. cereus* in particular, supplying six of the eight data points used to calculate it. As such, this may explain why the endospores of *B. cereus* were found to be less resilient to Far-UVC inactivation than their vegetative cell counterparts. Furthermore, this highlights challenges with the collation and comparison of data generated using similar experimental methods and the potential for a single publication to dramatically impact outcomes and conclusions. Worthy of note is that all publications included in the systematised review which tested the Far-UVC inactivation of vegetative cell and endospore forms within a single study found the endospore forms to be more resilient (*B. cereus*: [4,7]; *C. sporogenes*: [4]; *B. subtilis*: [3]).

### 4.4. Influence of Bacterial Cell Density on Susceptibility to Far-UVC Inactivation

Intuitively, the higher the cell density, the greater the resulting Far-UVC dose required to achieve complete inactivation. This has been demonstrated within the reviewed publications [40] and the presented experimental results (Figure 3). However, the potential effects of cell density on the efficiency of inactivation are less clear.

In higher cell densities, a decrease in bactericidal efficiency has been identified for other UVC light sources with peak outputs around 254 nm [65,66]. As of yet, however, little research has investigated this for Far-UVC light. Furthermore, given the differing bactericidal mechanisms employed by 254 nm and Far-UVC, the dose–response curves and their typical phenomena (e.g., lagging and tailing) may also differ.

The reduced bactericidal efficiency of light-based inactivation at higher cell densities has historically been attributed to the shielding or shadowing of cells [65,67,68]. Within the context of the systematised review, this phenomenon was referenced by Matafonova et al. [40]. In the experimental work, the effect of cell density on 1-Log_10_ ID produced largely inconclusive results, ranging from a strong negative correlation for *E. coli* (r = −0.701; *p* = 0.187) to a very strong positive correlation for *S. aureus* (r = 0.910; *p* = 0.032) (Figure 4). As such, based on the experimental work, no clear relationship between 1-Log ID and cell density was identified.

To examine the same relationship for data collected through the systematised review, the Pearson correlation was again used (Figure 6, Table 5). Only vegetative cells met the inclusion criteria. For four of the eight species (*B. cereus*, *L. monocytogenes*, *S. pyogenes* and *P. aeruginosa*), there was a strong or very strong positive correlation between the two variables. In all remaining cases the correlation was also positive, except in the case of *S. enterica* (r = −0.430; *p* = 0.717). The identified correlation reached statistical significance for half of the species. For all those that reached statistical significance, the correlations were moderately positive to very strong positive. These results suggest that efficiency of inactivation may decrease as cell density increases. Furthermore, of the eight species included here, five were assessed within the single study conducted by Matafonova et al. [40]. The authors concluded too that, for the numbers and species used, inactivation efficiency decreased with increased cell density and dramatically so at densities ≥10^6^ CFU·mL^−1^.

Regarding dose–response phenomena, the two focused on here were lagging and tailing. The presence of a shoulder in a dose–response curve has been considered the result of one of two possibilities: either a lag dose or lag time. In the case of the former, it is proposed that a threshold dose to initiate inactivation must be reached, before which little inactivation occurs; the latter proposes that there is a lag in time between initiation of irradiation and the resulting inactivation [69]. Tailing in bacterial inactivation is thought to result from several factors: the most resistant survivors requiring higher doses to achieve the same inactivation as the bacterial cells surrounding them [69]; the tendency of some cells and spores to form aggregates [70]; and the shadowing or shielding of cells by others overlying them [68].

Within the experimental results, a shoulder and tailing were most evident for *E. faecium* (Figure 3). This is likely due to its relative increased resistance to inactivation and, consequently, the greater observable detail afforded by its slower inactivation relative to dose. It is likely too, that, with sufficiently small dose intervals, such detail would also have been observed in the other species.

There was also some evidence of tailing in the experimental data. This was most obvious at higher cell densities. For example, for *E. faecium* in a cell density of 10^7^ CFU·mL^−1^, increasing the dose from 19.97 to 39.94 mJ·cm^−2^ increased the mean inactivation from 2.21-Log_10_ to 4.91-Log_10_. Yet, a further dose increase to 59.91 mJ·cm^−2^ only achieved a further 0.03-Log_10_ inactivation. Similarly, in a cell density of 10^5^ CFU·mL^−1^ for *S. aureus*, a dose of 9.99 mJ·cm^−2^ achieved a mean 3.18-Log_10_ inactivation, yet doubling that dose to 19.97 mJ·cm^−2^ only increased that mean inactivation by 0.82-Log_10_, without achieving complete measurable inactivation.

Yet, obvious tailing was not generally observed. Therefore, it can be concluded from the experimental work that although there is some evidence of tailing in the Far-UVC dose–response, it is not a dramatic feature of the results using the experimental setup employed here. Moreover, like the presence of a shoulder, it too appears to be more obviously present at higher cell densities. As previously highlighted, within the systematised review, Matafonova et al. [40] observed tailing in inactivation curves and also noted that this phenomena was accentuated by higher cell densities.

### 4.5. Influence of Irradiance on Susceptibility to Far-UVC Inactivation

The Bunsen–Roscoe law of reciprocity maintains that the photochemical reaction induced by irradiation is based solely on dose and is independent of irradiance and time [71]. Little practical research has been performed using UVC or Far-UVC specifically to test this theory. Furthermore, for other UV light sources, there are both supporting and dissenting accounts. Pousty et al. [72] found while using a wavelength of 265 nm to inactivate *E. coli*, that inactivation was independent of irradiance. However, they also found inactivation was irradiance-dependent for wavelengths of 285 and 295 nm. Yet, at 265 nm, Matsumoto et al. [73] recorded a bactericidal efficacy difference of an order of magnitude at a single dose by reducing the irradiance by 2–3 orders of magnitude. Contrary to the findings of Pousty et al. [72] at 285 and 295 nm, Matsumoto et al. [73] also found that inactivation was independent of the dose for a similar wavelength of 308 nm.

The experimental work performed in this study involved only a single irradiance and therefore could not be included in this section. Within the inclusion criteria of the systematised review, only a single publication employed more than one irradiance. Sugihara et al. [56] placed samples at two locations in a room at different distances from a Far-UVC light source, resulting in irradiance values of 0.001 and 0.0015 mW·cm^−2^. Consequently, there was insufficient review data collected from a single research study to gain proper insight into the effect of irradiance on the 1-Log_10_ ID.

However, when all systematised review literature was considered, seven species met the inclusion criteria, with *B. cereus* meeting it as both an endospore and vegetative cell. As such, there were three Gram-negative species and four Gram-positive species represented. In all but one case, there was a weak or negligible correlation between irradiance and the 1-Log_10_ ID. The exception was *S. enterica*, which had a strong positive correlation (r = 0.729) based on four data points. However, in none of the eight cases did the correlation reach statistical significance (*p* ≥ 0.148 for all).

These results suggest that the 1-Log_10_ ID and therefore inactivation efficiency for a species is independent of the irradiance used. While it is possible that this is true only over the irradiance range employed by the studies included here, the range was substantial, stretching from 0.001 to 5 mW·cm^−2^.

## 5. Conclusions

Several conclusions can be drawn from this study investigating the fundamental efficacy of Far-UVC bacterial inactivation in transparent minimal media. From both the experimental and systematised review work, a Gram-type effect on Far-UVC resilience was evident, with Gram-positive species generally requiring higher Far-UVC doses than Gram-negative species to achieve a similar level of inactivation. Although some inter-species and inter-genus differences in Far-UVC resilience were observed, the calculated mean* Far-UVC 1-Log ID was relatively low for both vegetative cell and endospores at 7.18 and 10.74 mJ·cm^−2^, respectively. Furthermore, it was shown that these doses are sufficient to achieve ≥1-Log_10_ inactivation in the majority of species included in this study. Regarding cell form, endospores in general displayed higher resilience to Far-UVC light than vegetative cells, as expected. Although there was some suggestion from the systematised review data that the efficiency of inactivation may decrease with increased cell density, this was not generally observed experimentally; further experimental work is required to accurately define this relationship. Finally, the Far-UVC dose–response curves from the experimental work generally displayed the typical phenomena seen in other UV dose–response curves of a shoulder and tailing. However, this was most evident at higher cell densities; smaller dose intervals than those employed here would likely accentuate these features.

In conclusion, this study has shown Far-UVC light to be an effective decontamination tool against a range of bacterial vegetative cells and endospores. Consequently, this technology may offer a valuable tool in the fight against AMR and hospital-associated infections. Furthermore, this study will act as a useful reference index for other researchers.

## Figures and Tables

**Table 1:** Inclusion and exclusion criteria employed in systematised review of literature.

Inclusion Criteria	Exclusion Criteria
Wavelengths ≥ 200, ≤230 nm	Wavelengths ≤ 199, ≥231 nm
Transparent liquid suspension	Multiple wavelengths used in combination
	Opaque or nutritious liquid suspension
	Bacteria on surfaces Bacteria in aerosols Pulsed light sources Cocktails of multiple bacteria
	Uncommon or genetically modified mutant strains

Note: Studies were included only in the categories for which all data were specified (i.e., if a publication included information on the irradiance employed experimentally but omitted the cell density, that paper was included only in the irradiance category).

**Table 2:** Relationship designation method followed for Pearson correlation coefficients, according to values outlined by Mukaka [38].

Pearson Correlation Coefficient	Relationship Designation
0.00 to 0.30	Negligible correlation
0.30 to 0.50	Weak positive or negative
0.50 to 0.70	Moderate positive or negative
0.70 to 0.90	Strong positive or negative
0.90 to 1.00	Very strong positive or negative

**Table 3:** Dose required for a 1-Log_10_ inactivation of bacterial species suspended in PBS at a range of cell densities, from 10^1^ to 10^7^ CFU·mL^−1^, using Far-UVC light at a delivered irradiance of 0.62 mW·cm^−2^. 1-Log_10_ ID value was calculated by dividing the dose (mJ·cm^−2^), at which the greatest statistically significant inactivation was achieved prior to tailing by the corresponding mean inactivation (Log_10_ CFU·mL^−1^) (based on the inactivation kinetics presented in Figure 3). From these experimental values, mean 1-Log_10_ ID values for Gram-positive and -negative species of 8.67 mJ·cm^−2^ and 4.30 mJ·cm^−2^ were calculated, respectively.

	Far-UVC 1-Log_10_ Inactivation Dose (mJ·cm^−2^)					
	10^1^ CFU·mL^−1^	10^2^ CFU·mL^−1^	10^3^ CFU·mL^−1^	10^5^ CFU·mL^−1^	10^7^ CFU·mL^−1^	Mean ± SD
*E. coli*	6.38	4.80	4.95	3.91	3.09	4.63 ± 1.23
*E. faecium*	17.16	10.88	7.95	7.87	11.30	11.03 ± 3.78
*P. aeruginosa*	3.11	2.05	3.09	5.90	5.66	3.96 ± 1.72
*S. aureus*	6.91	4.03	4.13	5.55	10.91	6.30 ± 2.83

**Table 4:** Far-UVC 1-Log_10_ ID value (mJ·cm^−2^) for all vegetative cells and endospores for which data were collected through systematised review. Where 1-Log_10_ ID needed to be calculated for an individual species, the dose (mJ·cm^−2^) at which the greatest statistically significant inactivation was achieved prior to tailing was divided by the corresponding inactivation (Log_10_ CFU·mL^−1^). For all data used to calculate median values, references are included; in some cases, multiple values were taken from a single publication.

Bacterial Species	Gram Type	Cell Form	Median Far-UVC 1-log_10_ ID (mJ·cm^−2^)	No. of Data Points (from One of More Studies)	References
*A. kookii*	Negative	Veg. cells	2.93	1	[39]
*B. cereus*	Positive	Veg. cells	49.31	8	[4,7,40]
*B. subtilis*	Positive	Veg. cells	4.68	10	[3,40,41,42]
*C. jejuni*	Negative	Veg. cells	1.02	1	[4]
*C. sporogenes*	Positive	Veg. cells	5.56	1	[4]
*E. mundtii*	Positive	Veg. cells	6.90	1	[39]
*E. coli*	Negative	Veg. cells	4.32	22	[4,39,40,41,43,44,45,46,47,48,49,50,51,52,53,54]
*L. pneumophila*	Negative	Veg. cells	1.67	1	[54]
*L. monocytogenes*	Positive	Veg. cells	1.71	4	[43,46,53,54]
*M. fortuitum*	Positive	Veg. cells	3.18	2	[41,42]
*M. smegmatis*	Positive	Veg. cells	10.45	1	[54]
*M. terrae*	Positive	Veg. cells	5.25	1	[55]
*Pantoea* spp.	Negative	Veg. cells	3.73	1	[42]
*P. aeruginosa*	Negative	Veg. cells	3.43	7	[4,7,41,42,45,46]
*P. fluorescens*	Negative	Veg. cells	2.01	1	[39]
*S. enterica*	Negative	Veg. cells	2.27	4	[4,43,53,54]
*S. aureus*	Positive	Veg. cells	4.31	20	[3,4,7,40,41,43,44,46,48,52,54,56]
*S. carnosus*	Positive	Veg. cells	3.28	1	[39]
*S. epidermidis*	Positive	Veg. cells	5.14	3	[45,57]
*S. hominis*	Positive	Veg. cells	4.85	1	[57]
*Stenotrophomonas* spp.	Negative	Veg. cells	3.73	1	[42]
*S. pyogenes*	Positive	Veg. cells	33.14	6	[40]
*Y. enterolytica*	Negative	Veg. cells	2.20	1	[50]
*A. acidoterrestris*	Positive	Endospores	6.97	1	[58]
*B. cereus*	Positive	Endospores	20.86	3	[3,4,7]
*B. pumilus*	Positive	Endospores	12.11	2	[59]
*B. subtilis*	Positive	Endospores	9.33	3	[3,47,60]
*B. thuringiensis* Al Hakam	Positive	Endospores	10.57	1	[3]
*C. difficile*	Positive	Endospores	9.62	3	[3,4]
*C. pasteurianum*	Positive	Endospores	2.40	1	[7]
*C. sporogenes*	Positive	Endospores	15.41	1	[4]
*S. griseus*	Positive	Endospores	6.38	1	[7]
*T. vulgaris*	Positive	Endospores	13.75	1	[7]

**Table 5:** Data describing the Pearson correlation between bacterial cell density and Far-UVC 1-Log_10_ ID for all bacterial species for which three or more different cell density values were available in the collected systematised review data. All bacteria are in vegetative cell form. Calculations performed using Minitab (v18) software (Minitab LLC, State College, PA, USA). * denotes significant *p*-value (*p* < 0.05).

Bacterial Species	No. of Data Points	No. of Contributing Papers	r-Value	Relationship	*p*-Value
*B. cereus*	8	3	0.807	Strong Positive Correlation	0.015 *
*B. subtilis*	8	3	0.529	Moderate Positive Correlation	0.177
*L. monocytogenes*	3	3	0.838	Strong Positive Correlation	0.367
*S. aureus*	16	10	0.491	Weak Positive Correlation	0.054
*S. pyogenes*	6	1	0.976	Very Strong Positive Correlation	0.001 *
*E. coli*	21	15	0.658	Moderate Positive Correlation	0.001 *
*P. aeruginosa*	7	6	0.989	Very Strong Positive Correlation	<0.001 *
*S. enterica*	3	3	−0.430	Weak Negative Correlation	0.717

**Table 6:** Data describing the Pearson correlation between irradiance and Far-UVC 1-Log_10_ ID for all bacterial species for which three or more irradiance values were available in the collected systematised review data. All bacteria are in vegetative cell form unless specified otherwise. Calculations were performed using Minitab (v18) software (Minitab LLC, State College, PA, USA). Significant Pearson Correlation did not result in any case (*p* < 0.05).

Species	No. of Data Points	No. of Contributing Papers	r-Value	Relationship	*p*-Value
*B. cereus*	8	3	−0.131	Negligible Correlation	0.757
*B. cereus* (endospores)	3	3	0.451	Weak Positive Correlation	0.702
*B. subtilis*	10	4	0.492	Weak Positive Correlation	0.148
*L. monocytogenes*	4	4	0.245	Negligible Correlation	0.755
*S. aureus*	19	11	0.097	Negligible Correlation	0.692
*E. coli*	21	15	−0.020	Negligible Correlation	0.930
*P. aeruginosa*	7	6	−0.181	Negligible Correlation	0.697
*S. enterica*	4	4	0.729	Strong Positive Correlation	0.271

## Data Availability

Data supporting this publication are stored by the University of Strathclyde. Details of the data and how they can be accessed are available from the University of Strathclyde KnowledgeBase at https://doi.org/10.15129/60aba21d-b58e-4617-baf4-79f48ebdb26d (accessed 24 July 2024).

## Supplementary Materials

The following supporting information can be downloaded at: https://www.mdpi.com/article/10.3390/pathogens13080698/s1, Table S1: Database information and search fields used in systematised literature review; Table S2: The search terms and Boolean operators used in the systematised literature review.
