# Supplementary material for: The Antibacterial Efficacy of Far-UVC Light: A Combined-Method Study Exploring the Effects of Experimental and Bacterial Variables on Dose–Response
Source: Pathogens. 2024 Aug 19;13(8):698. doi: 10.3390/pathogens13080698 (PMC11357679; doi:10.3390/pathogens13080698)
Supplement: Supplementary file 1 [file pathogens-13-00698-s001.zip › pathogens-3147893-supplementary.pdf]

The Antibacterial Efficacy of Far-UVC light: A Combined-Method Study Exploring the Effects of Experimental and Bacterial Variables on Dose-Response

MDPI Pathogens

David T Griffin[1,2], david.t.griffin@strath.ac.uk

Terence Gourlay[1], terence.gourlay@strath.ac.uk

Michelle Maclean[1,2,*], michelle.maclean@strath.ac.uk

[1]Department of Biomedical Engineering, University of Strathclyde, Glasgow G4 0NW, UK; [2]The Robertson Trust Laboratory for Electronic Sterilisation Technologies (ROLEST), Department of Electronic & Electrical Engineering, University of Strathclyde, Glasgow G1 1XW, UK.

*Corresponding author email: michelle.maclean@strath.ac.uk

Supplementary Material – Search Criteria Employed in Systematised Review

Contained in this file are the database information and search fields (Table S1) and the Boolean search terms (Table S2) used during data collection in the systematised literature review.

Table S1: Database information and specific search fields used for data collection in the systematised literature review performed on 23rd February 2024.

Database	Website	Search Field Used	No. Results
Compendex (via Engineering Village)	www.engineeringvillage.com	Abstract	237
Pubmed	www.pubmed.ncbi.nlm.nih.gov	Title/Abstract	121
Scopus	www.scopus.com	Abstract	461
		Total	819
	Total No. Results After Duplicate Removal		485
	Total No. Results After Non-English Language Removal		483
	Total After Screening		25

Table S2: Boolean search terms and operators used in Compendex, Pubmed and Scopus during data collection in the systematised literature review performed on 23rd February 2024.

**Search Term 1**		**Search Term 2**		**Search Term 3**		**Search Term 4**
Far-UVC Light		*Inactivation*		*Bacteria*		-Non-related content for exclusion-
**Synonyms**	**Operator 1**	**Synonyms**	**Operator 2**	**Synonyms**	**Operator 3**	**Synonyms**
excilamp OR medium-pressure OR "medium pressure" OR kr-cl OR krcl OR krypton-chloride OR krypton chloride OR far-uvc OR "far-ultraviolet*" OR "far ultraviolet*" OR "far uvc" OR "200 nm" OR "200-nm" OR "207 nm" OR 207-nm OR "220 nm" OR 220-nm OR "222 nm" OR 222-nm OR "225 nm" OR 225-nm OR "230 nm" OR 230-nm OR "233 nm" OR 233-nm	AND	inactivat* OR photoinactiv* OR inhibit* OR antimicrobial* OR harm* OR cytotoxi* OR antibacterial* OR impair* OR germicid* OR kill* OR disinfect* OR eradicat* OR peroxidat* OR susceptibil*	AND	microorganism* OR bacteri* OR bacteria* OR bacterio* OR endospore* OR spore* OR "vegetative cell" OR "vegetative cells" OR ESKAPE	NOT	peptide* OR "drug delivery" OR drug-delivery OR "drug target*" OR drug-target* OR nanomaterial* OR nanoparticle* OR nanocomposite* OR nanoemulsion*
